# Neuronal Correlates of Product Feature Attractiveness

**DOI:** 10.3389/fnbeh.2018.00147

**Published:** 2018-07-17

**Authors:** Franziska Schoen, Matthias Lochmann, Julian Prell, Kirsten Herfurth, Stefan Rampp

**Affiliations:** ^1^Division of Sports and Exercise Medicine, Department of Sport Science and Sport, Friedrich-Alexander-Universität Erlangen-Nuremberg, Erlangen, Germany; ^2^Department of Neurosurgery, University of Halle, Halle, Germany; ^3^Department of Neurosurgery, University Hospital Erlangen, Erlangen, Germany

**Keywords:** magnetoencephalography, electroencephalography, neurology, brain activity, attractiveness, emotion

## Abstract

Decision-making is the process of selecting a logical choice from among the available options and happens as a complex process in the human brain. It is based on information processing and cost-analysis; it involves psychological factors, specifically, emotions. In addition to cost factors personal preferences have significant influence on decision making. For marketing purposes, it is interesting to know how these emotions are related to product acquisition decision and how to improve these products according to the user's preferences. For our proof-of-concept study, we use magneto- and electro-encephalography (MEG, EEG) to evaluate the very early reactions in the brain related to the emotions. Recordings from these methods are comprehensive sources of information to investigate neural processes of the human brain with good spatial- and excellent temporal resolution. Those characteristics make these methods suitable to examine the neurologic process that gives origin to human behavior and specifically, decision making. Literature describes some neuronal correlates for individual preferences, like asymmetrical distribution of frequency specific activity in frontal and prefrontal areas, which are associated with emotional processing. Such correlates could be used to objectively evaluate the pleasantness of product appearance and branding (i.e., logo), thus avoiding subjective bias. This study evaluates the effects of different product features on brain activity and whether these methods could potentially be used for marketing and product design. We analyzed the influence of color and fit of sports shirts, as well as a brand logo on the brain activity, specifically in frontal asymmetric activation. Measurements were performed using MEG and EEG with 10 healthy subjects. Images of t-shirts with different characteristics were presented on a screen. We recorded the subjective evaluation by asking for a positive, negative or neutral rating. The results showed significantly different responses between positively and negatively rated shirts. While the influence of the presence of a logo was present in behavioral data, but not in the neurocognitive data, the influence of shirt fit and color could be reconstructed in both data sets. This method may enable evaluation of subjective product preference.

## Introduction

Consumer neuroscience is a novel research field aiming to investigate the subjective neuronal processes in response to marketing relevant stimuli (Lee et al., 2007). The main aim is to elucidate information about consumer preferences, which are either not consciously accessible or which may be biased by social and communicative factors. In contrast, conventional marketing methods, such as interviews and questionnaires, are thought to be significantly distorted by such factors.

Current literature covers many aspects of consumer neuroscience, ranging from product attractiveness, advertisement design to decision making and willingness to pay. An equally large number of putative direct and indirect neuronal markers have been suggested. Indirect markers utilize eye-tracking (Khushaba et al., 2013), galvanic skin conductance (GSR; Groeppel-Klein, 2005; Ohme et al., 2009), and heart rate measurements (Kenning and Linzmajer, 2011). In contrast, methods such as functional magnetic resonance imaging (fMRI), electro- and magneto-encephalography (EEG/MEG) evaluate brain activity directly, opening a window into the neuronal underpinnings and mechanisms.

In the presented study, we investigate neuronal correlates of product attractiveness independent of pricing and willingness to pay. We aimed at differentiating early cortical processing stages and identifying corresponding differences between positive and negative valuations. Such markers could be used for objective classification of subjective preferences in future applications e.g., for product development. Both MEG and EEG have excellent temporal and good spatial resolution and provide complementary perspectives on the same neuronal activity (Rampp and Stefan, 2007; Goldenholz et al., 2009; Ding and Yuan, 2013), allowing differentiation of activation sequences.

Current literature on MEG and EEG studies addressing emotion shows a trend away from the concepts of unique emotional centers to distributed networks (Doesburg et al., 2015). In a review, Kragel and LaBar (2016) emphasize the application of multivariate statistical tools to reconceptualize how emotion constructs might be embedded in large-scale brain networks, after prior localization approaches largely failed. Findings from pattern analyses of neuroimaging data show that affective dimensions and emotion categories are uniquely represented in the activity of distributed neural systems that span cortical and subcortical regions. D'Hondt et al. (2013) investigated the behavioral and cerebral response to peripherally presented affective stimuli using MEG. Arrows were preceded by peripherally presented emotional and neutral pictures. Subjects responded faster, when the orientation of the arrow was congruent with the location of the previously presented emotional scene. Non-predictive emotional peripheral information interfered with subsequent responses to foveally presented targets. That behavioral effect was correlated with an early (135 ms) increase in left orbitofrontal located cerebral sources. The authors suggest that the prior spatial distribution of emotional salience grabs attentional resources and influences the performance in the center of the visual field. In an EEG event-related-potential study, Goto et al. (2017) evaluated whether well-known neural markers of selective attention to motivationally-relevant stimuli were modulated by variations in subjective preference toward consumer goods in a virtual shopping task. They propose that early event-related potentials (ERPs, e.g., the N200) to consumer goods could be indicative of preferences driven by unconditional and automatic processes, whereas later ERPs such as the late positive potential (LPP) and positive slow waves (PSW) could reflect preferences built upon more elaborative and conscious cognitive processes. In a review about neuroaesthetics Cinzia and Vittorio (2009) summarize findings from recent neuroscientific studies about the aesthetic experience of visual artworks. Aesthetic experience is characterized by the activation of sensorimotor areas, core emotional (e.g., insula and amygdala) and reward-related centers (e.g., anterior cingulate cortex, orbitofrontal cortex). Kunkel et al. (2018) investigated evaluative processing of moral and emotional content during comprehension in an ERP-study. With visual sentence presentation varying in emotional and moral scenarios they showed that morality scenarios trigger a semantic-cognitive analysis, but affective evaluation when judging the emotional content. Phan et al. (2004) examined findings across 55 positron emission tomography (PET) and fMRI studies in a meta-analysis and aimed to determine patterns of activations in different emotions and across various emotional tasks. Findings suggest that several discrete brain regions are involved in specific emotions or emotional tasks, while others were more involved in general emotion perception/evaluation or regulation without regard to a specific emotional state. How these brain regions may be functionally connected in an “emotion network” in the human brain is still unknown and is an essential question for future studies.

EEG and MEG have been used to investigate responses to specific, marketing related stimuli, e.g., logos (Handy et al., 2010) or TV commercials (Astolfi et al., 2008; Vecchiato et al., 2011a,b). Thomas et al. (2013), Li et al. (2014), and Yilmaz et al. (2014) investigated the positive or negative evaluation of emotional words, food, cosmetics, and shoes. They could find significant differences in early, i.e., N100, N200, and P300 (Li et al., 2014) and later, i.e., 600–750 ms (Thomas et al., 2013) time intervals and in the lower frequency bands, i.e., 4–5 Hz (Yilmaz et al., 2014). Keuper et al. (2013) performed a combined MEG/EEG study and found early activation differences in the first 80–300 ms between positive and negative words in temporal and frontal language-related structures, as well as in occipital and parietal directed attention related regions. They conclude that different neuronal networks are active when positive vs. negative words are processed. They suggest “emotional tagging” of word forms during language acquisition. Such tagging would lead to different processing strategies, including enhanced lexical processing of positive words and a very fast language-independent alert response to negative words. Vecchiato et al. (2010, 2011b) found asymmetrical distribution of frequency specific activity in frontal and prefrontal areas, which are associated with emotional processing (Davidson and Irwin, 1999). “Like” conditions were correlated with increased theta and alpha oscillations in the left anterior hemisphere, while “dislike” conditions showed such increases on the right anterior side (Vecchiato et al., 2010, 2011b).

Such changes and markers could be used to evaluate the pleasantness of logos and product appearance by objective means, thus avoiding a subjective bias by customers who may not be able or willing to fully explain their choice. We conducted the presented proof-of-concept study investigating the effect of the different product features on brain activity to evaluate, whether these neuroscientific methods could be utilized as tools for marketing and product design. In this study sports shirts were the product of choice.

We specifically investigated whether the presence of the brand logo on sports shirts, as well as their color and fit, influence activation in general and frontal asymmetric activation specifically (Vecchiato et al., 2010, 2011b). We recorded brain activity with a 248-channel magnetometer and simultaneous 64-channel EEG in order to investigate the two following questions: (1) Are preferences for specific features (presence of the company logo, color, and fit) reflected by asymmetrical frontal activations? (2) Does the activation allow the evaluation of attractiveness, i.e., ranking of the specific product features, which could be used to optimize product design?

The main hypothesis is that EEG and MEG are suitable methods to evaluate questions in consumer neuroscience. The temporal resolution is optimal for visual processing and responses to pleasantness. We investigate the mechanisms of preferences and disfavor to products and product features in order to find a marker in space, time and frequency. The marker will then be used to build a classificator for the categories “like” and “dislike.”

## Materials and methods

### Participants

A total of 10 healthy participants (average age 22.1 ± 5.9) were selected. Inclusion criteria were determined according to the target group of the products of interest. Criteria were male gender, adults, active interest, and participation in sports, right-handedness, normal or corrected to normal vision, compatibility with MEG, i.e., no metal implants, etc., and no current neurological or psychiatric disorder.

### Ethics statement

This study was reviewed and approved by the Ethics Committee of the Medical Faculty, University Hospital Erlangen. All participants gave their informed written consent to participate in the study.

### Stimuli

**Figure 1:**
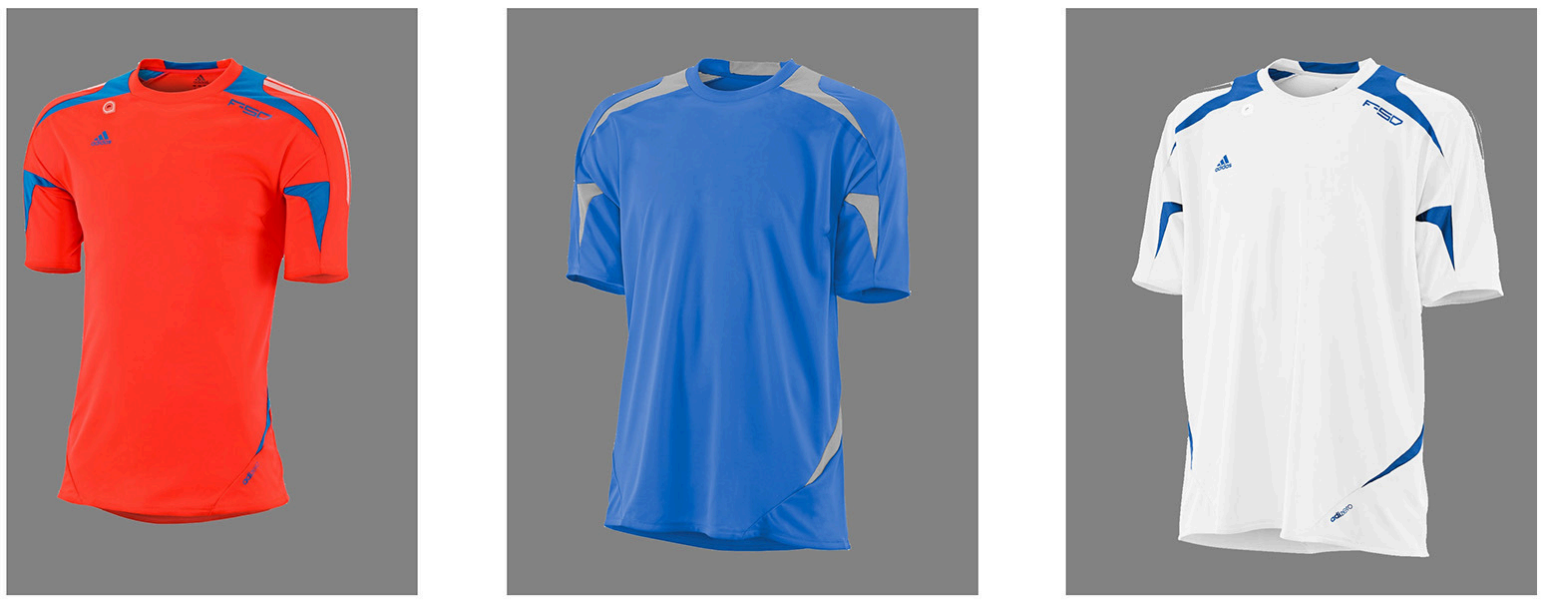
Soccer shirts as visual stimuli varying in color, fit, and presence of a branding.

Stimuli consisted of rendered images of soccer t-shirts presented on a light gray background. The brightness of the background was controlled to avoid glaring. The variables of interest were chosen regarding to the most salient features of a shirt. All shirts had the same basic design, however differed in respect to fit (tight, medium, and wide), color (white, orange, blue), and presence of a small “adidas” logo (present or not present). This resulted in 18 different combinations. Figure 1 shows exemplarily an orange shirt in tight fit, with branding, a blue shirt in medium size, without branding, and a white shirt in wide fit, again with branding.

### Procedure

Individual EEG electrode positions were recorded using a 3D digitizer stylus (Polhemus, Colchester, VT, USA). Head positions were registered using five head coils at the beginning and the end of each individual recording run. Subjects were positioned in the MEG dewar in a seated position and images were presented via video projector and a mirror and screen system.

**Figure 2:**
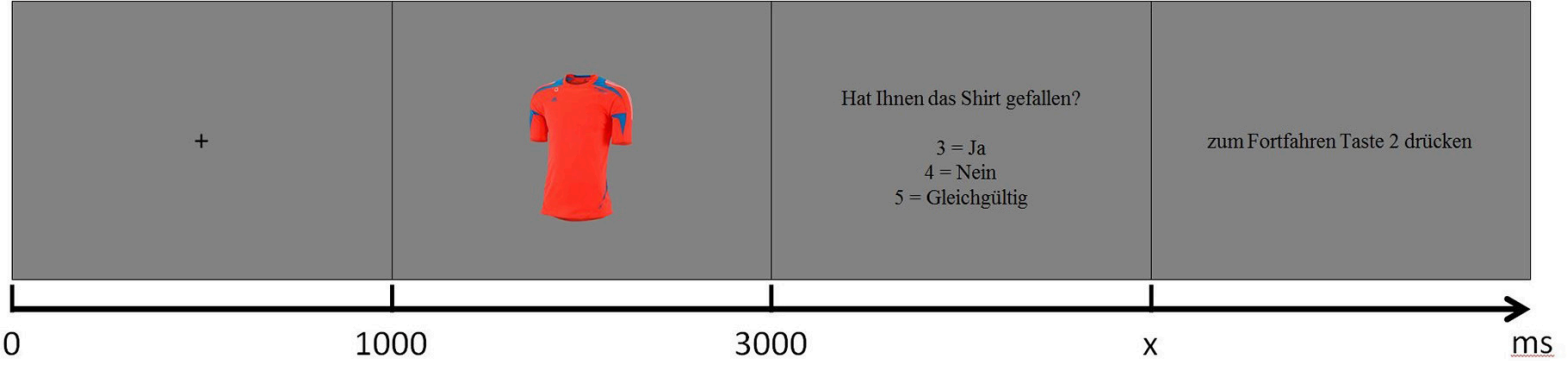
Experimental design. At first subjects saw a fixation cross for 1 s, followed by the shirt picture for 2 s. Then the subjects had to decide if they like or dislike the product or if they don't care (“Did you like the shirt? 3 = yes, 4 = no, 5 = don't care”). To proceed they had to press button 2. In the last two steps the subjects were allowed to blink.

Subjects were instructed to evaluate each shirt, presented in randomized order, in regard to general attractiveness and provide their subjective impression after a cue. Participants chose between like, dislike and don't care responses using a keyboard response box. A wider scale would have been preferable and would have potentially enabled more robust statistics. It was however explicitly not chosen in order to minimize the time which was available to consider or reconsider the initial choice. The experimental design is illustrated in Figure 2. The paradigm was implemented using E-Prime software (Psychology Software Tools, Sharpsburg, PA, USA). The complete procedure with 720 trials was subdivided into four runs of 180 trials each. Participants were allowed to pause between runs as much as needed.

### MEG/EEG recordings

MEG data was recorded with a 248-channel magnetometer Magnes 3600 WHS-system (4D Neuroimaging, San Diego, CA, USA). Simultaneous 64-channel EEG, electrooculography (EOG) to detect eye movements and blinking and electrocardiography (ECG) to record the heart rate were recorded using an ANT-Neuro amplifier (ANT Neuro, Enschede, The Netherlands). EEG electrodes were mounted on a lycra cap (ANT Neuro, Enschede, The Netherlands) according to the extended 10–10 system including a temporo-basal ring. Impedance was kept below 5 kΩ. A sampling rate of 508.63 Hz, as well as a 0.1–100 Hz online filter was applied.

### MEG/EEG analysis

**Figure 3:**
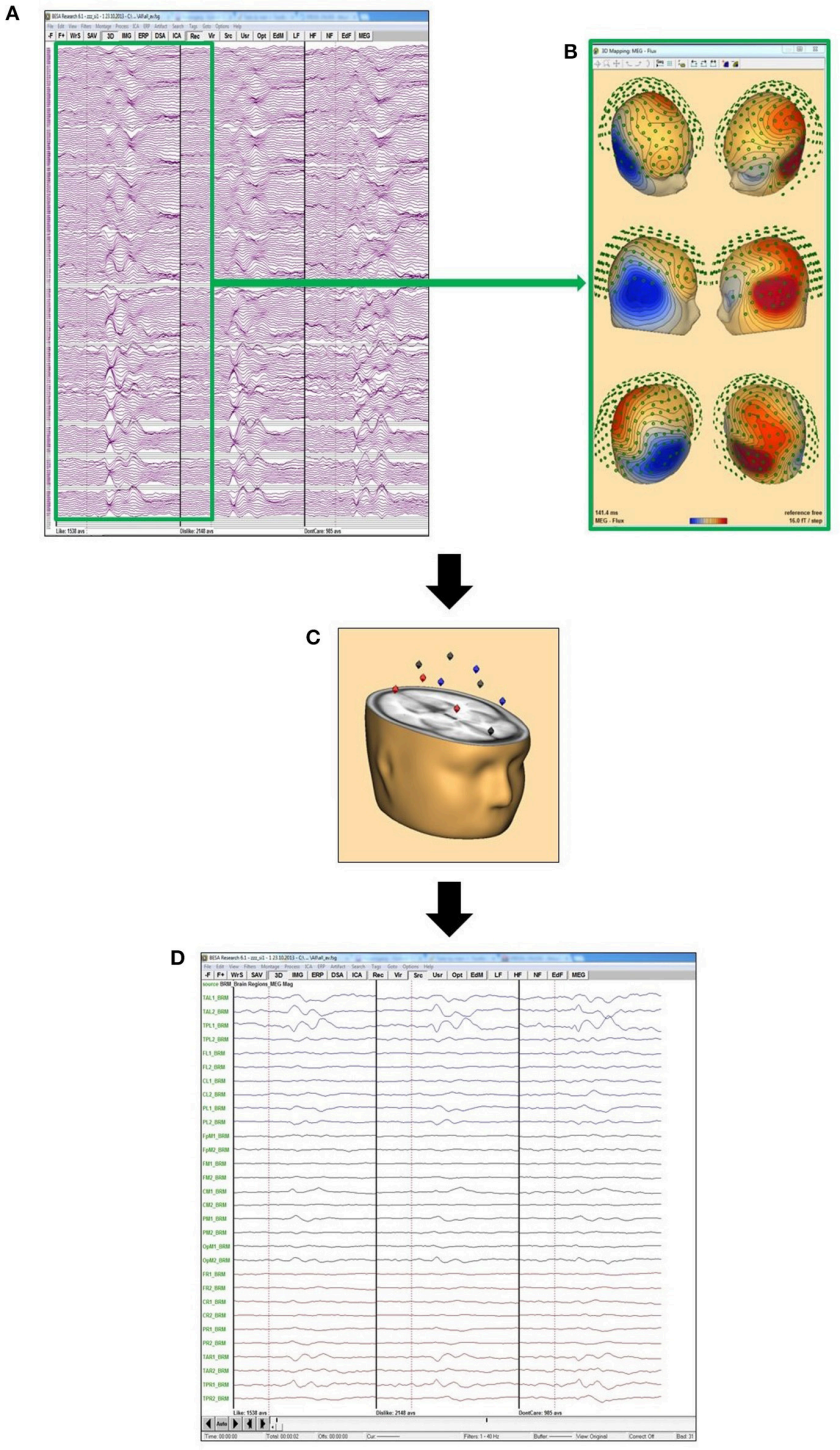
Source montage concept: **(A)** grand averages of “like” (left), “dislike” (middle), and “don't care” for all MEG channels; **(B)** MEG flux map exemplarily for the maximum of the “like” condition, calculated from the grand average “like” data from **(A)**; **(C)** this data is projected on the 29 BESA standard source space positions; **(D)** data projected on the positions in **(C)** to evaluate the activation in these 29 different brain areas.

Eye artifacts, as well as ECG components in both EEG and MEG were removed using an adaptive artifact correction method implemented in BESA Research 6.0 (BESA GmbH, Gräfeling, Germany) (Ille et al., 2002), as well as source montages for projection of sensor data to 29 source space positions (Figure 3). Trials were excluded from analysis, when EEG or MEG amplitudes exceeded 120 μV or respectively, 3,000 pT after artifact correction and 1 Hz high pass filtering. Single channels were excluded if considerable artifacts occurred in most trials. Interpolation to an 81 electrode standard montage was performed to retain comparability for sensor level analysis also in cases with such excluded channels. Intraindividual MEG/EEG averages were generated for each of several conditions: like, dislike and don't care response, as well as each individual shirt feature combination. Grand averages were calculated weighting each subject according to the number of trials used for the individual average.

### Statistics

Statistic consisted of several steps. If not stated otherwise, analysis was performed on 1–40 Hz filtered data, based on the results of the frequency spectrum step below. Statistics were calculated using BESA Statistics 1.0 (BESA GmbH, Gräfeling, Germany), Matlab R2011b (The Mathworks, Natick, MA, USA), and SPSS 20 software (IBM, Armonk, NY, USA).

#### Behavioral response analysis

Participants' responses concerning evaluation of the presented shirts were analyzed using analysis of variance (ANOVA) with factors fit, color and presence of a logo. *Post-hoc* tests of significant main effects were evaluated using *t-*test and Bonferroni correction. Corrected *p-*values are reported. Significant interactions were evaluated qualitatively.

#### Frequency spectrum

**Figure 4:**
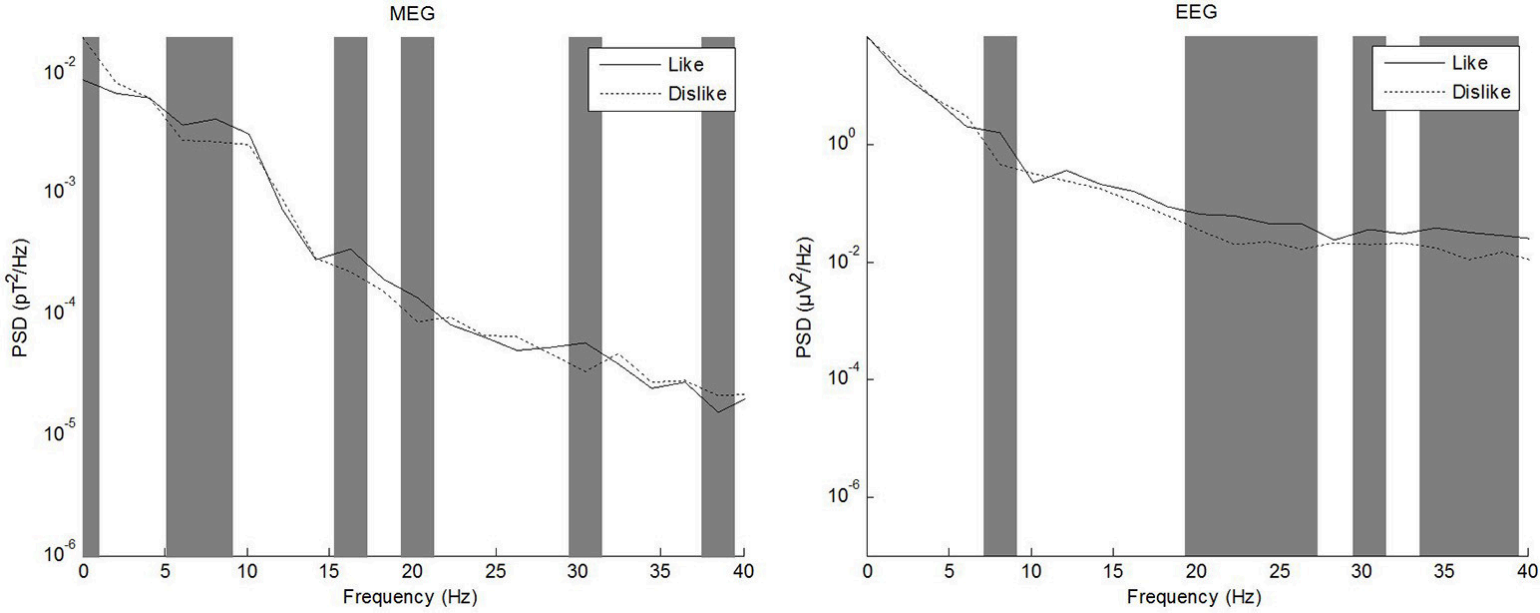
Power spectral density of MEG **(left)** and EEG **(right)** grand averages for the conditions like and dislike.

In the literature, different frequency ranges have been explored in regard to processing of emotional stimuli and subjective evaluation of attractiveness. Vecchiato et al. (2010, 2011a,b) have repeatedly demonstrated utility of theta and alpha frequency bands, while e.g., Keuper et al. (2013) investigated a wider band of 1–40 Hz. We thus calculated frequency spectra of source level data using a Fast Fourier Transform (FFT) as a first step to evaluate whether differences between conditions were apparent in the complete band of 1–40 Hz or whether further analysis should be constrained to e.g., theta or alpha bands. To this end, power spectral density (PSD) was calculated for each data channel and condition using ~2 Hz frequency bins. PSD values of each frequency bin were then compared separately between conditions using a paired *t-*test. The sample size for each test therefore was equal to 2 conditions ^*^ number of channels (EEG: 64, MEG: 248). The resulting *p*-values (one per frequency bin) were corrected for multiple comparisons using the false-discovery rate procedure according to Benjamini and Hochberg (1995). Corrected *p-*values below 0.05 were considered significant. EEG and MEG were evaluated separately. The frequency range of interest for subsequent analyses was evaluated based on the comparison of like vs. dislike conditions. Figure 4 shows PSD for MEG and EEG comparing the conditions like and dislike.

#### Sensor level analysis of like vs. dislike conditions

Intraindividual sensor level averages were subjected to a non-parametric permutation test (Bullmore et al., 1999; Ernst, 2004; Maris and Oostenveld, 2007), contrasting like vs. dislike conditions. EEG and MEG were evaluated separately, using 1,000 permutations.

#### Source space analysis

a) Comparison of neuronal and behavioral responses

For comparison of behavioral responses regarding preferences of individual samples/shirts (Table 1) and neuronal EEG/MEG correlates, we used data projected to the 29 source space ROIs only. We then compared the shirt-specific grand averages to the grand averages of like and dislike conditions by means of pearson correlations. The rationale of our analysis was that similar behavior should be accompanied by similar neuronal patterns, i.e., in this specific context, that positively evaluated shirt combinations should be reflected by patterns similar to the “like” grand average. Thus, the “like” grand average was used as an internal reference pattern. Corresponding to the 18 samples (shirt combinations), 18 like correlation values resulted, representing a neuronal estimate for the degree of positive subjective valuation (“like similarities”). For evaluation of this method and the underlying assumptions, we calculated the correlation of these like similarities with the behavioral response, i.e., specifically the percentage of positive valuations (Table 1).

b) Influence of features on neuronal responses

**Table 1:** Behavioral responses of subjects to individual feature combinations, given as the mean percentage of all single responses (branding = 1; no branding = 0).

**Fit**	**Color**	**Branded**	**Like (%)**	**Dislike (%)**	**Don't care (%)**	**Favorite**
Tight	White	0	67	17	16	
Tight	Orange	0	48	22	30	
Tight	Blue	0	67	24	9	1
Tight	White	1	78	11	11	2
Tight	Orange	1	59	18	23	3
Tight	Blue	1	75	18	7	6
Medium	White	0	27	36	37	
Medium	Orange	0	20	54	26	
Medium	Blue	0	22	50	28	
Medium	White	1	44	33	23	
Medium	Orange	1	29	52	19	
Medium	Blue	1	36	41	23	
Wide	White	0	20	54	26	
Wide	Orange	0	17	62	22	
Wide	Blue	0	11	62	26	
Wide	White	1	22	59	19	
Wide	Orange	1	25	60	15	
Wide	Blue	1	18	54	27	

“*Favorite” column holds times the combination was selected as favorite. Subjects were allowed to name 1 or 2 favorite combinations*.

Influence of shirt features (fit, color, and branded) on like similarities were investigated. The analysis was constrained on specific time segments and regions of interest, based on previous literature (Keuper et al., 2013): P1 (80–120 ms) in left temporal and frontal areas (source montage projection channels FL, TAL), P2 (150–180 ms) in frontal areas (FL, FM, FR), and EPN (200–300 ms) in occipital, parietal and posterior cingulate areas (PL, PM, PR, OpM) Influence of shirt features on the subjective attractiveness were evaluated by performing an ANOVA (with factors fit, color and branded) on “like similarities.” Although it is to be expected that these similarity values do not show a normal distribution, e.g., due to the low sample size, ANOVA was still used due to robustness of ANOVA to violation of the normality assumption (Glass et al., 1972; Harwell et al., 1992; Lix et al., 1996). Analysis of interactions was limited to two levels due to the low sample size.

## Results

### Behavioral response analysis

The tight white shirt with logo collected the most like responses during recordings, followed by the same combination in blue. ANOVA of behavioral data revealed main effects for all three factors (Table 2). A significant interaction was only identified for the factors fit^*^color. *Post-hoc* tests for all significant factors showed that subjects preferred tight to medium (*p* < 0.001) and wide fit (*p* < 0.001), and also liked medium more than wide fit (*p* = 0.006). In regard to color, white was the top choice and was preferred significantly more often than orange (*p* = 0.007), while blue was an intermediate choice which differed in the number of “like” responses from both white and orange only on the level of a tendency (*p* = 0.088 white, *p* = 0.073 orange). Regarding to the presence of a logo, subjects significantly preferred branded shirts. The interaction between the factors fit and color showed that the combination of tight fit with blue color was the favorite combination of these two factors, while blue shirts with wide fit yielded the fewest like responses.

**Table 2:** ANOVA of behavioral responses.

**Source**	**Sum sq**.	**d.f**.	**Mean Sq**.	***F***	**eta^2^**	**Sig**.
Corrected model	130603.39	13	10046.415	97.328		**0**
Constant term	408306.72	1	408306.722	3955.609	0.757	**0**
Fit	113398.11	2	56699.056	549.291	0.210	**0**
Color	4841.44	2	2420.722	23.452	0.009	**0.006**
Branded	6766.72	1	6766.722	65.555	0.013	**0.001**
Fit ^*^ color	4906.22	4	1226.556	11.883	0.009	**0.017**
Fit ^*^ branded	676.78	2	338.389	3.278	0.001	0.144
Color ^*^ branded	14.11	2	7.056	0.068	0.000	0.935
Error	412.89	4	103.222		0.001	
Total	539323.00	18				
Corrected total variation	131016.28	17				

*Significant interactions marked as bold values*.

### Frequency spectrum

Frequency spectrum calculation from source level grand averages of like, dislike, and don't care conditions revealed prominent differences between conditions in the whole frequency band up to 40 Hz. Qualitative differences between modalities were especially pronounced in delta and alpha frequency ranges. Based on these results, we decided not to restrict analysis to only theta or alpha bands.

### Sensor level analysis of like vs. dislike conditions

#### EEG (Figure 5)

Sensor level comparison of like vs. dislike conditions revealed five clusters of significant differences: Cluster 1 (*p* < 0.0001) between 350–370 and 405–412 ms in left temporo-occipital areas, cluster 2 (*p* = 0.006) between 465 and 535 ms in central areas, cluster 3 (*p* = 0.018) between 205 and 270 ms in right parieto-occipital areas, cluster 4 (*p* = 0.019) between 390–400 and 415–435 ms in right parietal areas and cluster 5 (*p* = 0.019) between 480 and 520 ms in right fronto-temporal areas.

**Figure 5:**
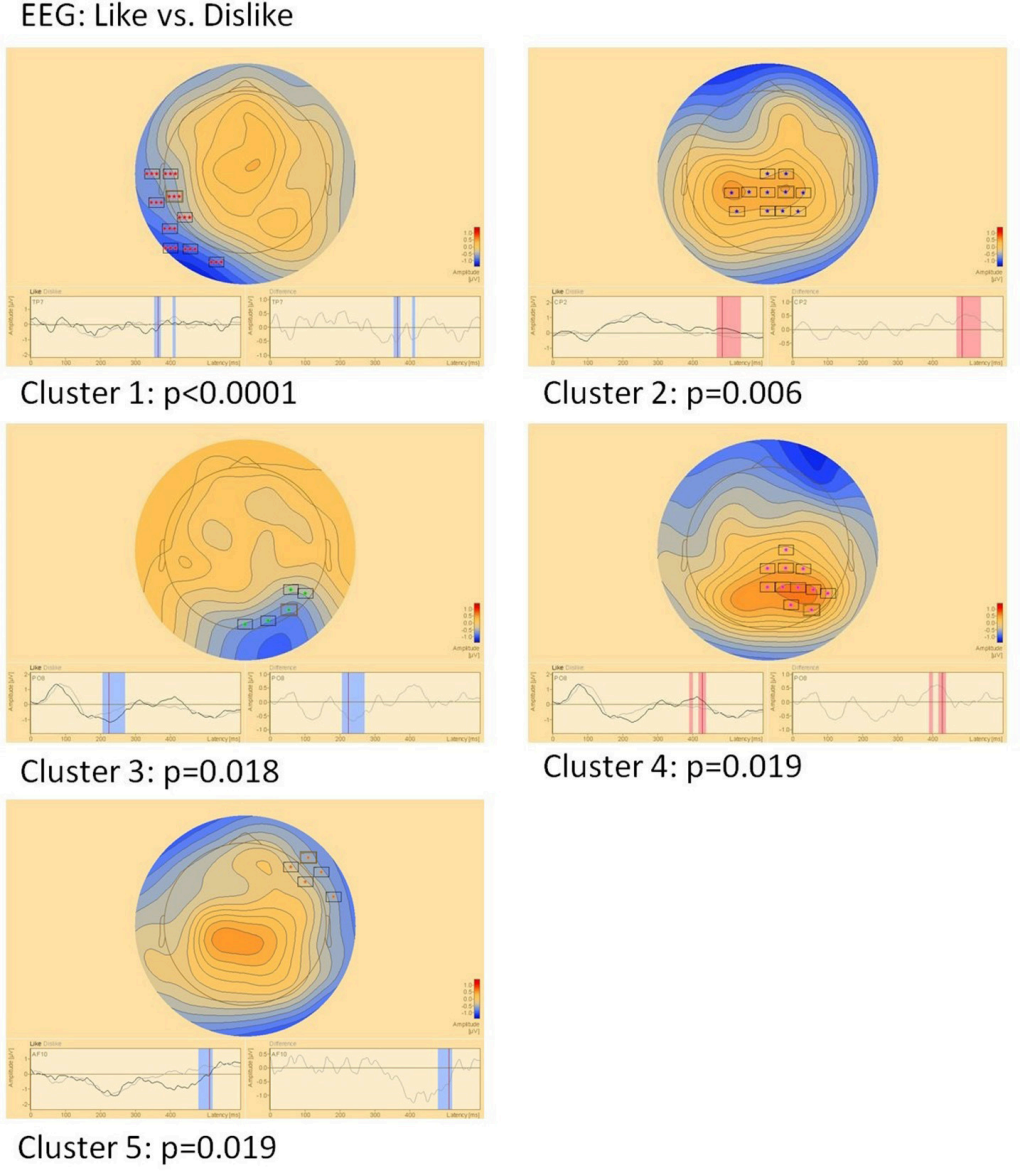
Clusters of significant differences in EEG sensor level analysis of like vs. dislike conditions. Boxes mark sensors with significant differences.

#### MEG (Figure 6)

In MEG two clusters of significant differences were found: Cluster 1 (*p* = 0.028) between 120 and 145 ms in right parietal areas and cluster 2 (*p* = 0.037) in the same location, however later between 160 and 180 ms. Location but not timing of MEG clusters correspond well to EEG clusters 3 and 4.

**Figure 6:**
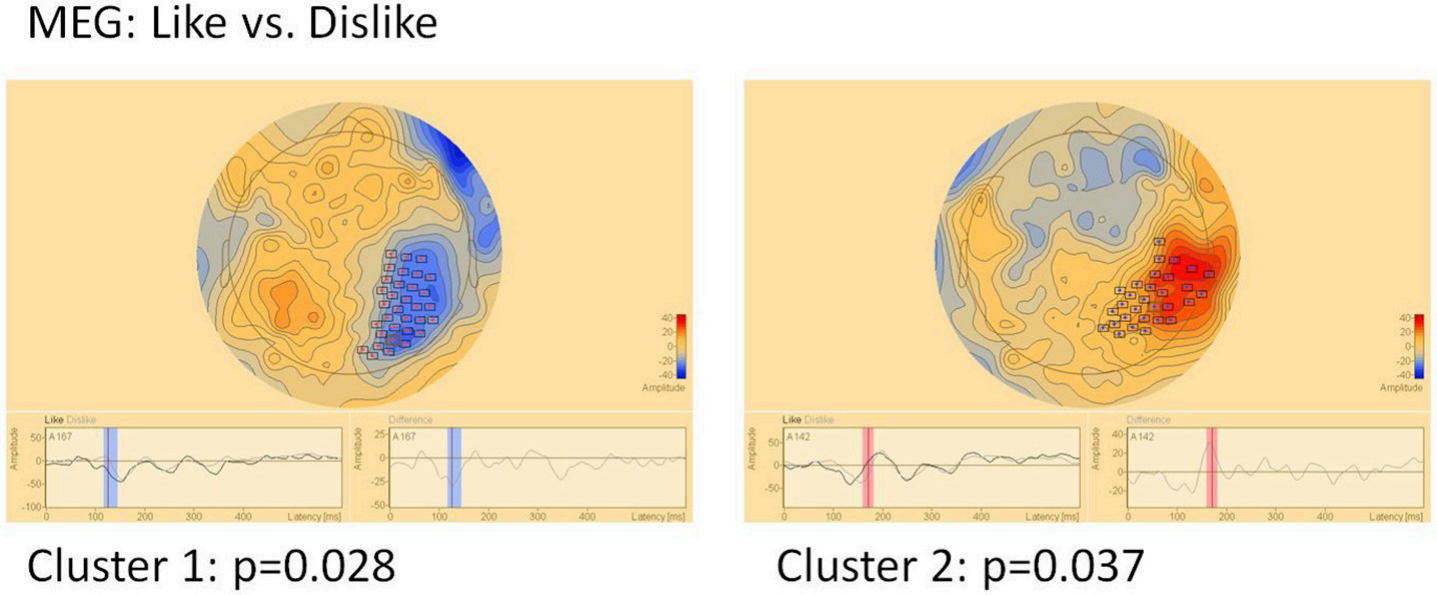
Clusters of significant differences in MEG sensor level analysis of like vs. dislike conditions. Boxes mark sensors with significant differences.

### “Like similarity” as a marker for subjective valuation

The degree of positive behavioral evaluation was compared to the “like similarity” maker as a neuronal correlate. Pearson correlation analysis showed a correlation of *r* = 0.69 (*p* = 0.0015) using the complete post-trigger MEG-data and *r* = 0.72 (*p* = 0.0007) using EEG. The correlation of MEG and EEG “like similarity” was *r* = 0.43 on the statistical level of a tendency (*p* = 0.08). Combining the MEG and EEG markers by means of canonical correlation yielded *r* = 0.84 (*p* < 0.0001).

### Evaluation of feature characteristics on source activity

#### EEG

Source space analysis of EEG showed no clear influence of shirt features on like similarity in P1 and P2 time segments and ROIs. In EPN, however, influences could be observed, which reached the statistical level of a tendency for fit (*p* = 0.053) and presence of a logo (*p* = 0.098). Based on the results of sensor level analysis additional segment/ROIs were investigated, Ce1–Ce5, corresponding to timing and topography of EEG clusters 1–5. Here, influences on the levels of significant tendencies could be identified in Ce1 and Ce5 for color (*p* = 0.076) and presence of a logo (*p* = 0.083), respectively. Table 3 shows the ANOVA results for like similarity in EEG.

**Table 3:** ANOVA for like similarity in EEG.

**Factor**	**Sum sq**.	**d.f**.	**Mean sq**.	***F***	**eta^2^**	**Sig**.
**P1: 80–120 ms, LEFT TEMPORAL AND FRONTAL AREAS**						
Fit	0.0126	2	0.0063	0.07	0.008	0.9364
Color	0.27266	2	0.13633	1.43	0.178	0.2774
Branded	0.1056	1	0.1056	1.11	0.069	0.3134
Error	1.14422	12	0.09535		0.745	
Total	1.53508	17				
**P2: 150–180 ms, FRONTAL AREAS**						
Fit	0.13528	2	0.06764	2.74	0.234	0.1047
Color	0.11294	2	0.05647	2.29	0.196	0.144
Branded	0.03278	1	0.03278	1.33	0.057	0.2716
Error	0.29627	12	0.02469		0.513	
Total	0.57727	17				
**EPN: 200–300 ms, OCCIPITAL, PARIETAL, AND POSTERIOR CINGULATE AREAS**						
Fit	0.00813	2	0.00406	3.8	0.323	0.0528
Color	0.00076	2	0.00038	0.35	0.030	0.7097
Branded	0.00344	1	0.00344	3.21	0.137	0.0984
Error	0.01285	12	0.00107		0.510	
Total	0.02518	17				
**Ce1: LEFT TEMPORO-BASAL (TPL)**						
Fit	0.44657	2	0.22329	0.8	0.077	0.4716
Color	1.79617	2	0.89808	3.22	0.310	0.0759
Branded	0.21018	1	0.21018	0.75	0.036	0.4023
Error	3.34622	12	0.27885		0.577	
Total	5.79914	17				
**Ce5: LEFT TEMPORO-BASAL (TPL)**						
Fit	0.27125	2	0.13562	1.55	0.146	0.2525
Color	0.21695	2	0.10848	1.24	0.117	0.3247
Branded	0.31469	1	0.31469	3.59	0.170	0.0825
Error	1.05211	12	0.08768		0.567	
Total	1.85501	17				

#### MEG

Source space analysis of MEG showed an early significant influence of the fit feature on like similarity (*p* = 0.046) in the P1 time segment and area, i.e., between 80 and 120 ms in left temporal and frontal areas. The P1 like similarity correctly identified a tight fit as the favorite choice (mean 0.53, max 0.79), followed by medium (mean 0.24, max 0.51) and wide (mean 0.21, max 0.45).

Shirt color demonstrated a significant influence in P2, i.e., between 150 and 180 ms in bilateral frontal areas. Orange color showed the highest like similarity values at P2 (mean 0.80, max 0.91), followed by white (mean 0.72, max 0.86), and by blue (mean 0.60, max 0.79).

The presence of a logo did not show any significant influence in P1, P2, or EPN. In EPN, no significant influence of any feature could be observed.

Based on the results of sensor level analysis, i.e., MEG clusters 1 and 2, an additional segment/ROI, termed Cm was investigated: 120–180 ms in right parietal areas (PR, CR, PM) and revealed an additional effect of color features (*p* = 0.0025). Here, considerably higher like similarity values more closely reflected the subjects' behavioral preference: Blue was the favorite color (mean 0.87, max 0.94), followed by orange (mean 0.75, max 0.84) and white (mean 0.71, max 0.84). Table 4 shows the ANOVA results for like similarity in EEG.

**Table 4:** ANOVA for like similarity in MEG.

**Factor**	**Sum sq**.	**d.f**.	**Mean sq**.	***F***	**eta^2^**	**Sig**.
**P1: 80–120 ms, LEFT TEMPORAL AND FRONTAL AREAS**						
Fit	0.36102	2	0.18051	4.04	0.372	**0.0455**
Color	0.05656	2	0.02828	0.63	0.058	0.5478
Branded	0.01614	1	0.01614	0.36	0.017	0.559
Error	0.53614	12	0.04468		0.553	
Total	0.96986	17				
**P2: 150–180 ms, FRONTAL AREAS**						
Fit	0.03634	2	0.01817	1.22	0.106	0.3285
Color	0.12566	2	0.06283	4.23	0.368	**0.0407**
Branded	0.00097	1	0.00097	0.07	0.003	0.803
Error	0.17827	12	0.01486		0.522	
Total	0.34123	17				
**P3: 200–300 ms, OCCIPITAL, PARIETAL, AND POSTERIOR CINGULATE AREAS**						
Fit	0.00314	2	0.00157	0.33	0.042	0.7284
Color	0.00314	2	0.00157	0.33	0.042	0.7284
Branded	0.00002	1	0.00002	0	0.000	0.9564
Error	0.05798	12	0.00483		0.778	
Total	0.07456	17				
**CM: 120–180 ms, RIGHT PARIETAL AREAS**						
Fit	0.01099	2	0.0055	0.49	0.029	0.6264
Color	0.23247	2	0.11624	10.29	0.613	**0.0025**
Branded	0.00002	1	0.00002	0	0.000	0.9707
Error	0.13558	12	0.0113		0.358	
Total	0.37906	17				

*Significant interactions marked as bold values*.

## Discussion

Our results show clear differences between cortical activity in like vs. dislike conditions. Furthermore, influence of specific product features could be observed.

### Frequency spectrum

Most interesting frequency bands for studying neuronal networks regarding emotions are mainly delta (0.5–4 Hz) and theta (4–8 Hz) bands. Alpha oscillations (8–14 Hz) classically reflect cortical inhibition and get are reduced following sensory stimulus presentation (Doesburg et al., 2015). Some activations are also visible in beta (13–30 Hz) and gamma bands (30–50 Hz). Gamma band activity however is difficult to evaluate using non-invasive methods due to interference by s also reflect artifacts and electromyogram (EMG). As said before, trends in neuroscience go to the neuronal networks and frequency couplings. Doesburg et al. (2015) explain how coordinated cross-frequency and inter-regional oscillatory cortical dynamics underlie typical and atypical brain activation, and the formation of distributed functional ensembles supporting cortical networks underpinning sensation and perception. The alpha-theta-gamma (ATG) switch is one result of their explanations: local regional activation by an external stimulus via sensory pathways entails attenuated alpha and increased theta and gamma activity and increased interactions among theta and gamma rhythms. Our results are only partially overlapping with the results of Vecchiato et al. (2010, 2011a,b). While they concentrated on theta and alpha frequency bands, spectral analysis of our data revealed significant differences in frequency ranges up to 40 Hz. This observation is more in line with the work of Keuper et al. (2013), who investigated processing of hedonic quality of emotional words in EEG and MEG frequency bands between 1 and 40 Hz. Furthermore, involved frequency bands could and likely depend on stimulus features and modalities used to present them. Vecchiato et al. (2010) for example, investigated responses to TV commercials, thus stimulation utilized vision (including movement perception) and hearing in contrast to only visual presentation of shirt images in our study. A study from Yilmaz et al. (2014) investigated spectral characteristics as indicators of consumer preferences. In their 19-channel EEG study, subjects had to evaluate different pictures of shoes. While the highest discrimination power was found in the theta band, significant differences between like and dislike conditions were also observed in frequency ranges up to 40 Hz. Literature regarding other aspects of consumer neuroscience, like willingness to pay or decision making show similar results. Differences might be explained by differences in the underlying cortical processes, such as calculations or considerations regarding the price or benefits. Attractiveness in contrast is supposed to rely on the first emotional impression (like or dislike). Ramsøy et al. (2018) for example report frontal asymmetry also in beta and gamma bands during a willingness to pay task, but not in the alpha band. Here the subjects had to choose an amount of money that they were willing to pay for a product they had seen before. Frontal asymmetry in the alpha band was found to predict consumers choice in the face of changes in price and brand provided (Ravaja et al., 2013). In a study evaluating movie trailers, individual preferences and the movies population-wide commercial success are predicted (Boksem and Smidts, 2015). These predictions are based on midfrontal beta for individual preferences and gamma oscillations clustered around frontocentral areas to predict the population-wide success.

### Patterns of cortical responses

While EEG/MEG responses to attractiveness evaluation of shirts did not clearly show the frontal asymmetry postulated by previous studies (Vecchiato et al., 2010, 2011a,b), distinctive differences of cortical activity between positively and negatively evaluated shirts could be observed. While component Ce5 showed a frontal asymmetric topography with significant differences limited to the right side, all other EEG and MEG differences occurred in other areas with some pronunciation of central, parietal, and temporal areas. Most such components were close to the midline with a tendency to be observable on the right side. Parietal activations were also found by other studies (e.g., Bröckelmann et al., 2011, 2013; Rehbein et al., 2011). It is noteworthy that e.g. Bröckelmann et al. (2013) found that parietal activation is especially enhanced when positive stimuli are processed. This is in line with the strong association of activity in this area with the degree of attractiveness and liking of color features in the MEG Cm component. Keuper et al. (2013) similarly describe enhanced occipital activation after positive stimuli, which could correspond topographically with EEG component Ce3 (note that the sign of the EEG or MEG amplitude in sensor data is of little significance in regard to the overall activation quantity but is instead tied to the orientation of the underlying generators). Posterior parietal and occipital EEG/MEG field maps are also concordant to findings of Keil et al. (2002), who showed that differences between affective evaluation of emotional and neutral pictures in contrast to Keuper et al. (2013), is reflected by differing activation patterns in the visual association cortex.

Experiments conducted by Vecchiato et al. (2010, 2011a,b) repeatedly show frontal asymmetric activity as a maker for valuation of stimuli. Alternatively, such frontal activation could also be caused by increased attention, when subjectively relevant, i.e., positive or negative stimuli are presented. An area activated in case of increased attention is the anterior cingulate cortex (ACC, Carretié et al., 2004). Li et al. (2014) could also find early activations in anterior cingulate cortex (ACC) area in an attention allocation task with emotional words. Differing ACC activation is likely associated and would be in line with changes and asymmetry of EEG potentials over the frontal lobe. This would provide a further explanation for the lack of a comparable finding in our data, as our paradigm was rather long and repetitive and may elicit increased attention and thus ACC activity only to a limited degree.

### Influence of product features

Using a novel attractiveness marker, the “like similarity,” behavioral evaluation of both fit and color of shirts could be reproduced in specific spatio-temporal components of neuronal processing. Effects of fit and subjective attractiveness appeared early (80–120 ms) and exactly reflected the behavioral sequence, while color influenced responses in a later time range (150–180 ms). The response to color could reproduce blue as the favorite choice in Cm. It is however noteworthy that a total of two cortical components could be identified (P2, Cm), which show a significant influence of color, but different ranking. These results apparently show that, at least in the setting of our study, color preference is less unambiguous than fit features, reflected in cortical markers.

In contrast to the clear behavioral data, straightforward cortical correlates of the subjective evaluation of branded vs. unbranded shirts could not be identified. Only influences on the significance level of a tendency could be observed in EEG, which point to posterior and temporo-basal areas, in line with form processing systems in the visual association cortex. Subjects commented that the size of the logo was too small and bad to see on the projected pictures. In further studies the size of the logo should possibly be increased to find more meaningful effects. Effects of branding could be demonstrated in other studies. Thomas et al. (2013) compared brand and no-name cosmetics and food products in a go/no-go association task intermixed with positive and negative words in EEG. The subjects were instructed to classify pictures (food/cosmetics) and words (positive/negative word) separately. Differences between the congruent (brand and positive word) and incongruent (brand and negative word) conditions were found in the late positive component (600–750 ms). They were enhanced for brand stimuli in contrast to no-name products and seem to hint at the existence of implicit attitudes.

### Methodology

For statistical comparisons, we deliberately did not evaluate all possible combinations of time segments and ROIs, but instead utilized previously reported definitions or hypotheses taken from sensor level analysis. This procedure was chosen to avoid pitfalls of multiple comparison testing and the subsequent increase of false positive findings. Incorporation of further neuroscientific findings about the interplay of visual processing, attention, and hedonic evaluation may provide candidates for better spatiotemporal analysis windows, possibly supported by explorative studies. The same seems advisable in regard to analyzed frequency bands, especially as there are varying suggestions in the literature (e.g., Vecchiato et al., 2011b vs. Keuper et al., 2013 and our own results).

“Like similarity” as analysis method was conceived for the present study. The rationale is based on the idea of an internal reference. That is, similar responses (to individual feature combinations) are thought to be similar to a “prototype” activation under like or dislike conditions. While the technique provided results consistent with behavioral data (preference of specific features) and literature (areas influenced by feature categories), robustness and validity should be further studied, e.g., by using simulation or strong affective stimuli.

EEG and MEG provided complementary information. While EEG showed more time segment and ROIs with significant differences, MEG provided a clearer view on influence of individual features. Especially in superficial cortical areas, MEG has a higher signal to noise ratio, while EEG provides a better, however still less than ideal depth sensitivity (Goldenholz et al., 2009). Furthermore, magnetic fields (in contrast to electric potentials) are almost undistorted by conductivity differences. These characteristics could explain the better like similarity values and clearer ANOVA results. EEG however, if optimal electrode caps are used, provides a better coverage of especially the inferior and basal areas. While MEG can certainly record activity from these areas, optimal positioning of the subject's head in the dewar may be difficult in practice in some cases.

### Future improvements

Both our results and performance of methodology suggests further development toward an automatic “black box” classificator of subjective attractiveness. Activity in certain spatiotemporal ROIs could be extracted automatically and used as inputs. This classificator could be trained, i.e., internal weights would be adjusted, to maximize the contrast between like and dislike conditions and output an estimate of the subjective attractiveness. A classificatory approach could provide a common basis and evaluation framework for various EEG/MEG analysis techniques and enable combination of different methods. Due to the limited sample size, contrast, and statistical power the results of ANOVA show limited robustness and can be considered as exploratory.

## Author contributions

FS, KH, and SR made substantial contributions to conception, design, acquisition of data, analysis and interpretation of data. ML and JP participated in the conception of the study, discussions and interpretation of results, drafting the article and final proofreading of the version to be submitted.

### Conflict of interest statement

FS and data recording were supported by the adidas AG. The funder had the following involvement with the study stimulus design and general objective. The remaining authors declare that the research was conducted in the absence of any commercial or financial relationships that could be construed as a potential conflict of interest.
